# Dielectric Characterization of Protonated Chitosan-Lignin Biocomposite Membranes: Influence of Chitosan and Lignin Types

**DOI:** 10.1021/acs.biomac.5c01090

**Published:** 2025-08-26

**Authors:** Mark H. Wolf, Nagore Izaguirre, Jalel Labidi, Amparo Ribes-Greus

**Affiliations:** a Research Institute for Materials Technology, 16774 Universitat Politècnica de València (UPV), Camino de Vera, s/n, 46022 Valencia, Spain; b Chemical and Environmental Engineering Department, Faculty of Engineering of Gipuzkoa, 83067 University of the Basque Country (UPV/EHU), Plaza Europa 1, 20018 Donostia, Spain

## Abstract

Biobased chitosan-lignin composite membranes with tailored dielectric and conductive properties were developed using chitosan of high (CS_H_) and low (CS_L_) molecular weight and degree of deacetylation, combined with kraft (KL) and organosolv lignin (OL) as fillers. The membranes were protonated by immersion in 1.0 M sulfuric acid. CS_H_ composites exhibit stronger ionic interactions with sulfate groups compared to CS_L_ composites, resulting in a dense structure that hinders water absorption and increases fragility. Chitosan interactions with sulfuric acid and lignin restrict the mobility of dielectric relaxations, with KL having a more pronounced effect than OL due to its smaller size and higher phenolic OH content. The membranes act as electrical insulators, exhibiting electron conductivities ranging from 10^–15^ to 10^–8^ S/cm between −10 and 170 °C, and proton conductivities between 2.9 × 10^–3^ and 4.4 × 10^–3^ S/cm at 60 °C. These properties make them promising candidates for use as biobased electrolytes in fuel cell applications.

## Graphical abstract

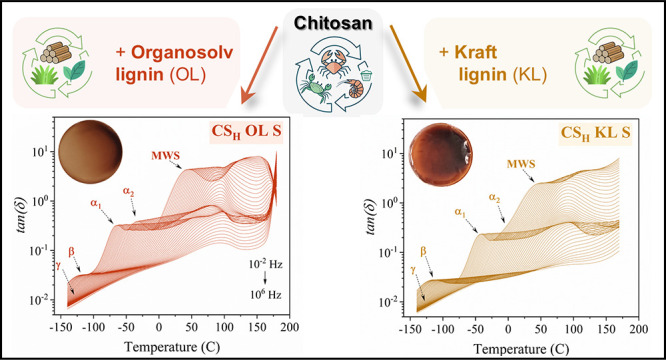

## 1 Introduction

The depletion and rising costs of petrochemical resources, as well as their significant environmental damage, have spurred growing interest in biobased materials. Natural polymers derived from renewable sources, such as chitin and lignin, provide eco-friendly alternatives to synthetic polymers due to their remarkable abundance, affordability, and biodegradability.^1^

Chitosan, the partially deacetylated form of chitin, is widely found in crustaceans, mollusks, insects, algae, and fungi.^2^ It can be extracted from residual seafood industry waste, which yields an economically valuable material while also preserving resources and reducing waste deposition.^3^ Chitosan′s abundant amine and hydroxy groups enable an easy way to tailor its properties through chemical modifications, cross-linking, or incorporation of fillers.^4,5^

Lignin, a major component of wood alongside cellulose and hemicellulose, can be obtained as a byproduct from the pulp and paper industry.^6^ Its properties are primarily influenced by its natural source and extraction process, with common methods including kraft pulping and organic solvent treatments.^7^ Lignin is commonly used as a reinforcing filler in chitosan-based materials, where it enhances mechanical properties, thermal stability, and acid resistance.^8–11^ The combination of these two natural polymers, both of which can be derived from industrial waste, contributes to sustainability by reducing the dependence on petroleum-based resources and creating high-value biocomposites.

To fully explore the potential applications of biobased composites, it is crucial to understand the detailed interactions between the polymers that constitute these materials. Dielectric thermal analysis (DETA) is a powerful technique for characterizing the dielectric relaxation spectrum and providing insights into the molecular interactions within composite systems.^12^

In a recent study, the conductive properties of chitosan-lignin biocomposites were enhanced by immersing them in a 1.0 M sulfuric acid solution.^13^ This approach is a common method for protonating polymer electrolytes, including the widely used perfluorosulfonic ionomer Nafion.^14,15^ Additionally, the sulfuric acid treatment alters the hydrophilicity of the composites and creates ionic interactions between polymer chains, improving structural stability and controlling water-induced swelling.^16–18^ To optimize chitosan-lignin biocomposites as materials for energy-related applications, it is essential to investigate how sulfuric acid solution immersion affects their dielectric relaxation spectra.

The aim of this work is to study the dielectric and conductive properties of different chitosan-lignin biocomposites modified by immersion in a sulfuric acid solution. Two types of chitosan were used: one with a higher molecular weight and higher deacetylation degree (CS_H_) than the other (CS_L_). Additionally, two lignin fillers, derived from kraft pulping (KL) and organosolv acetic extraction (OL) of eucalyptus chips, were employed to prepare biocomposites with distinct functional properties. The chemical structure of the modified biocomposites was assessed using Fourier-transform infrared (FTIR) spectroscopy. Water immersion studies were conducted to evaluate differences in water uptake and dimensional swelling. Thermal phase transitions were investigated through differential scanning calorimetry (DSC). Ultimately, dielectric thermal analysis (DETA) was used to study the dielectric relaxation spectrum, as well as the proton and electron conductivity of the biocomposite membranes. Structural variations among the modified composites and their intermolecular interactions were found to influence the thermal activation and cooperativity of their relaxation mechanisms, as well as their conductive properties.

## 2 Experimental Section

### 2.1 Materials and Reagents

Pulping kraft liquor and chips from eucalyptus were kindly provided by Papelera Guipuzcoana de Zikuaga (Hernani, Spain). Chitosan with a molecular mass (*M*_*w*_) of 100–300 kDa and a degree of deacetylation (DDA) of ≥75% (CS_L_), as well as chitosan with an *M*_*w*_ of 600–800 kDa and a DDA of ≥90% (CS_H_), were purchased from Thermo Scientific (Waltham, MA, USA). Acetic acid (glacial), formic acid (85%), and sulfuric acid (96%) were obtained from PanReac Química (Barcelona, Spain). Ethanol (absolute, ExpertQ) was purchased from Scharlab (Barcelona, Spain). Deionized water (<0,1 μS/cm) was obtained from an Autwomatic purifier from Wasserlab (Barbatáin, Spain).

### 2.2 Lignin Extraction, Preparation, and Characterization

First, the eucalyptus chips were ground to a size of 2 mm with a SM 100 Cutting Mill from Retsch (Haan, Germany). The humidity for future calculations was determined in triplets with an IR-60 moisture analyzer from Denver Instruments (New York, NY, USA). Then, the lignin was extracted from 100 g of the chips with a CIMV process in an autoclave type VAPOR-Line Lite II from VWR (Radnor, PA, USA) at 105 °C for 2.5 h, according to Labauze and Benjelloun-Mlayah.^19^ An acetic acid/formic acid/water mixture (55/30/15 wt %) was used for the extraction with a solid/liquid ratio of 1/20. After the reaction, the product was filtered (Nylon, 0,45 μm) and washed with the organosolv acetic solution.

Subsequently, the organosolv lignin from eucalyptus (OL) was precipitated from the solution with ∼3 times the volume of deionized water and left to stand overnight. The Kraft lignin (KL) was precipitated from the black liquor with sulfuric acid until the pH reached 2.0. The pH was controlled with a pH meter type Sension+ PH3 from Hach (Loveland, CO, USA), which was previously calibrated. Both lignin types were filtered (Nylon, 0.22 μm) with a high-pressure filtration system from Sartorius (Göttingen, Germany) and washed with deionized water until the pH reached neutral. Afterward, the lignin types were dried in an oven at 50 °C for 48 h, ground with a quartz mortar, and stored in sealed containers. The characterization of both lignin types, including FTIR spectroscopy, pyrolysis analysis, gel permeation chromatography (GPC), and UV–vis spectroscopy, is provided in the Supporting Information.

### 2.3 Preparation of Chitosan-Lignin Biocomposite Membranes by Solvent Casting

Both lignin types were dissolved in ethanol (*c* = 3 g/L) and stirred with an RT 10 magnet stirrer from IKA (Staufen, Germany) for 24 h. Afterward, they were filtered (Nylon, 0.22 μm) with a Sartorius filtration system to avoid aggregates in the final membranes. The two chitosan powders (CS_L_ and CS_H_) were each dissolved in 1% (v/v) aqueous acetic acid solution with a concentration of 10 g/L and stirred with a magnet stirrer type MR Hei-Tec from Heidolph (Schwabach, Germany) for 2 days. The lignin solution was added to the chitosan solution with a percentage of 10% (w/w) with respect to chitosan and homogenized with an ULTRA-TURRAX T25 disperser from IKA at 13,000 rpm for 5 min. Subsequently, the mixtures were degassed for 20 min in an ultrasonic bath type Elmasonic S 70 H from Elma (Singen, Germany) and cast into Petri dishes with a diameter of 14 cm. The solutions (200 mL) were horizontally placed in an incubator type Unimax 1010 from Heidolph at 25 °C for 2–3 days until the solvent completely evaporated. The membranes were stored in sealed plastic bags until further use.

### 2.4 Immersion in a Sulfuric Acid Solution

The chitosan-lignin composite membranes were protonated by immersion in a 1.0 M sulfuric acid (H_2_SO_4_) solution at 30 °C for 1 h. Afterward, the samples were cleaned with distilled water until the excess acid was removed and a neutral pH was reached, and then dried between glass plates under vacuum in a Heraeus (Hanau, Germany) vacuum oven VT 6025 at 40 °C for 24 h. The membranes that were immersed are labeled with an “S” at the end of their designation.

### 2.5 Physicochemical Characterization

#### 2.5.1 Fourier-Transform Infrared (FTIR) Spectroscopy

The FTIR spectra of the chitosan-lignin composites were recorded with a Nicolet iS50 FTIR Spectrometer from Thermo Scientific. The membranes were placed on the iS50 ATR accessory and measured in the wavelength range from 4000 to 500 cm^–1^. 32 scans per spectrum were collected with a spectral resolution of 4 cm^–1^. Prior to each series of samples, background spectra were recorded. For each material, five spectra at different positions of the sample were taken, and an average spectrum was calculated. The spectra were normalized at the band of the ring stretching of the polysaccharide structure at 900 cm^–1^.

#### 2.5.2 Water Immersion Studies

Water immersion studies were performed to assess the water uptake and swelling behavior of the biocomposite membranes. Therefore, three specimens of each membrane were immersed in distilled water at 30 °C for 48 h, and the changes in mass and thickness were calculated. Prior to immersion, the samples were dried under vacuum at 30 °C for 48 h to determine their dry mass (*m*_dry_) and thickness (*l*_dry_). The thickness was measured using a Mitutoyo (Kawasaki, Japan) measuring stand (model 215–611, ± 1 μm) at five different positions on each sample. After the immersion period, the samples were removed from the water and gently wiped with paper tissue before measuring their mass and thickness (*m*_wet_, *l*_wet_). The percentual water uptake (*M*_*t*_) and swelling degree (*S*_*t*_) were calculated using eqs 1 and 2, respectively.

$$M_{t}\left(\%\right)=\frac{\left(m_{\mathrm{wet}}-m_{\mathrm{dry}}\right)}{m_{\mathrm{dry}}}\times 100$$ 1

$$S_{t}\left(\%\right)=\frac{\left(l_{\mathrm{wet}}-l_{\mathrm{dry}}\right)}{l_{\mathrm{dry}}}\times 100$$ 2

#### 2.5.3 Differential Scanning Calorimetry (DSC)

The calorimetric phase transitions of the biocomposite membranes were assessed by using the Mettler-Toledo DSC 820 setup (Columbus, OH, USA). The DSC measurements were conducted under an inert nitrogen atmosphere at a flow rate of 50 mL/min. Samples weighing between 3 and 5 mg were placed in perforated 40 μL aluminum crucibles. The scanning program consisted of three consecutive heating–cooling–heating segments at a rate of 10 K·min^–1^: the first heating scan ranged from 25 to 140 °C, followed by a 20 min isothermal step. This was followed by a cooling scan to 25 °C and a subsequent second heating scan to 225 °C. The analysis of the calorimetric thermograms was performed with Mettler-Toledo’s STARe software (9.01).

#### 2.5.4 Dielectric Thermal Analysis (DETA)

The dielectric and conductive properties of the biocomposite membranes were obtained using a broadband dielectric impedance spectrometer from Novocontrol Technologies GmbH & Co. KG (Montabaur, Germany). The sample membranes (Ø = 20 mm) were sandwiched between two stainless-steel plates and placed in a BDS-1200 standard cell from Novocontrol. The temperature of the isothermal measurements was controlled using the Quatro Cryosystem (Δ*T* < 0.1 K). The dielectric data were obtained with the Alpha-A mainframe analyzer, with up to 10 points per frequency decade. Processing of the dielectric data was performed with the WinFIT software (3.4).

Two distinct DETA measurements were performed in an inert nitrogen (N_2_) atmosphere: (i) one to determine the dielectric relaxation spectrum and electron conductivity of dry sample membranes between −140 and 180 °C in 10 °C steps, within the frequency range from 10^–2^ to 10^7^ Hz, and (ii) another to assess the proton conductivity at 60 °C under hydrated conditions, from 10^0^ to 10^7^ Hz.

### 2.6 Theory Section

The dielectric relaxation spectra of the chitosan-lignin biocomposites were analyzed by means of the real part of permittivity (*ε′*), the imaginary part of permittivity (*ε″*), and the dielectric dissipation factor (*tan­(δ)*). The ε″*-*spectra of the composite membranes at every temperature were adjusted to Havriliak–Negami functions to determine the mode relaxation time (*τ*_max_) from the peak of each dielectric process.^20^ In the case where multiple relaxation processes overlapped, the Charlesworth method was applied for their deconvolution.^21^

The temperature dependence of molecular dynamics was studied by plotting the relaxation frequency (*f*_max_ = 1/(2 × π × *τ*_max_) against the inverse temperature in an Arrhenius map. Linear processes were adjusted to the Arrhenius model, while nonlinear processes were described using the Vogel–Fulcher–Tammann–Hesse (VFTH) model, as expressed in eqs 3 and 4.^22^

$$f_{\max}=f_{0}\times \exp\left(\frac{-E_{a}}{R\times T}\right)$$ 3

where *f*_0_ is a pre-exponential factor, *E*_*a*_ is the apparent activation energy, and *R* is the universal gas constant (*R* = 8.314 J·K^–1^·mol^–1^).

$$f_{\max}=f_{0}\times \exp\left(\frac{-D\times T_{V}}{T-T_{V}}\right)$$ 4

where *T*_V_ is the Vogel temperature, and *D* is the dynamic fragility parameter.

The macromolecular origin of the relaxation mechanisms in the composites was analyzed using the Starkweather–Eyring theory at 1 Hz, as described by eq 5.^23^

$$E_{a}=R\times T\times \left[22.92+\ln\left(T\right)\right]$$ 5

In this model, relaxations near the zero-entropy line have an intramolecular origin and lack significant cooperative interactions with neighboring entities. In contrast, relaxations of intermolecular origin involve interactions with neighbors, resulting in an increased inactivation energy.

The electronic conductivity of the membranes was studied by adjusting the low-frequency plateau of the real part of the conductivity (*σ*′) to Jonscher’s power law, as described in eq 6:^24^

$$\sigma^{\prime }\left(\omega \right)=\sigma_{dc}+A\omega^{n}$$ 6

Here, *σ*_DC_ represents the direct current (DC) conductivity component, while *A*ω^*n*^ corresponds to the alternating current (AC) conductivity component. In this expression, *A* is a pre-exponential factor, and *n* is a frequency exponent (0 < *n* ≤ 1).

The proton conductivity (*σ*_prot_) of the biocomposite membranes under hydrated conditions was calculated by using eq 7.

$$\sigma_{\mathrm{prot}}=\frac{l}{A\times R_{0}}$$ 7

where *l* is the distance between the electrodes, determined by the membrane thickness, *A* is the area of the electrodes, and *R*_0_ is the bulk resistance. The value for *R*_0_ was determined from the real part of impedance (*Z*′) at the point where the phase angle is closest to zero (Φ → 0).^25^

## 3 Results and Discussion

### 3.1 Structural Characterization of the Chitosan-Lignin Biocomposite Membranes

The chemical structures of the different chitosan-lignin biocomposites are assessed by FTIR spectroscopy. Figure 1 shows the normalized infrared spectra of the composite membranes after modification by immersion in a sulfuric acid solution. The protonated composite membranes show various absorption bands in the mid-infrared range, which are summarized in Table 1, along with their assignation.

**Figure 1:**
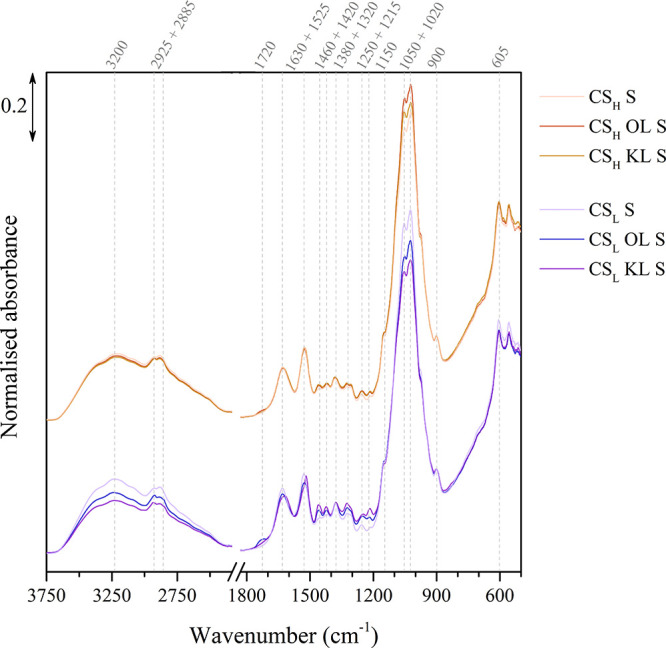
Normalized infrared spectra of the chitosan-lignin composite membranes after immersion in a sulfuric acid solution.

**Table 1:** Infrared Absorption Bands of the Protonated Chitosan-Lignin Biocomposites and Their Designation

wavenumber (cm^–1^)	band designation	assignation	reference
3200	O–H/N–H stretching	chitosan + lignin	^18,26,35,27–34^
2925 + 2885	C–H stretching of CH_2_ + CH_3_	chitosan + lignin	^18,26,28–30,33–35^
1720	C=O unconjugated stretching	lignin	^36–42^
1630	C=O stretching (amide I)	chitosan	^26–35^
1525	N–H deformation (amine and amide II)	chitosan	^18,26,35,27–34^
1460	CH_2_ stretching	chitosan + lignin	^36,37,40–43^
1420	symmetric deformation CH_3_	chitosan + lignin	^30,35,44^
1380	CH_3_ deformation of acetamide	chitosan	^33–35,45^
1320	syringyl ring breathing with C–O stretching	lignin	^36,40–42^
1250	C–O stretching vibration	chitosan	^28,35^
1215	C–C and C–O stretching	lignin	^36,37,40,43^
1150	C–O–C asymmetric stretching	chitosan	^18,26,27,30,34,35^
1050 + 1020	C–O stretching	chitosan	^18,26,27,30,33–35^
1030	–SO_3_ stretching	sulfate groups	^16,29,46^
900	polysaccharide ring stretching	chitosan	^30,44^
605	–S–O deformation	sulfate groups	^17,32^

The CS_H_ composites, which contain chitosan with a higher molecular mass (*M*_*w*_) and a higher degree of deacetylation (DDA), show lower infrared absorbance at 2925, 1630, and 1380 cm^–1^, due to fewer acetamide groups in the polysaccharide chain. Consequently, the greater availability of amino groups in these CS_H_ composites leads to a higher incorporation of sulfate ions (SO_4_^2–^) upon sulfuric acid solution immersion, as indicated by slightly increased absorption bands at 1030 and 605 cm^–1^. Additionally, the CS_H_ composites exhibit slightly higher absorbance between 2500 and 3500 cm^–1^, corresponding to various O–H and N–H stretching vibrations, due to the increased hydrophilicity of the polar amino groups and greater water uptake.

The composites containing lignin show their characteristic absorbance bands at 1460, 1320, and 1215 cm^–1^, which can also be appreciated in the infrared spectra of the lignin fillers in Figure S1. Additionally, the absorbance of the broad band around 3200 cm^–1^ decreases with lignin addition, as its nonpolar nature reduces the hydrophilicity of the composite.

Differences between the composites containing organosolv lignin (OL) and kraft lignin (KL) are evident at the unconjugated C=O band at 1720 cm^–1^, which appears mainly in OL due to the esterification of the propane chain during formic acid treatment.^36,47^ The composites containing KL show slightly lower absorbance for the sulfate ion bands at 1030 cm^–1^ compared to those with OL. Due to its smaller size and higher phenolic OH group content (see Tables S4 and S5 in the Supporting Information), KL distributes more evenly in the membrane and forms stronger interactions with the amino groups of chitosan.^48^ As a result, fewer amino groups are available to form Coulombic interactions with the sulfate ions upon immersion, resulting in lower incorporated quantities.

### 3.2 Water Uptake and Swelling of Composite Membranes

The water uptake (*M*_48h_) and swelling (*S*_48h_) behavior of the biocomposites are assessed by monitoring the mass and dimensional changes through immersion in water. Figure 2 shows the changes in the protonated chitosan-lignin membranes after immersion in distilled water for 48 h at 30 °C.

**Figure 2:**
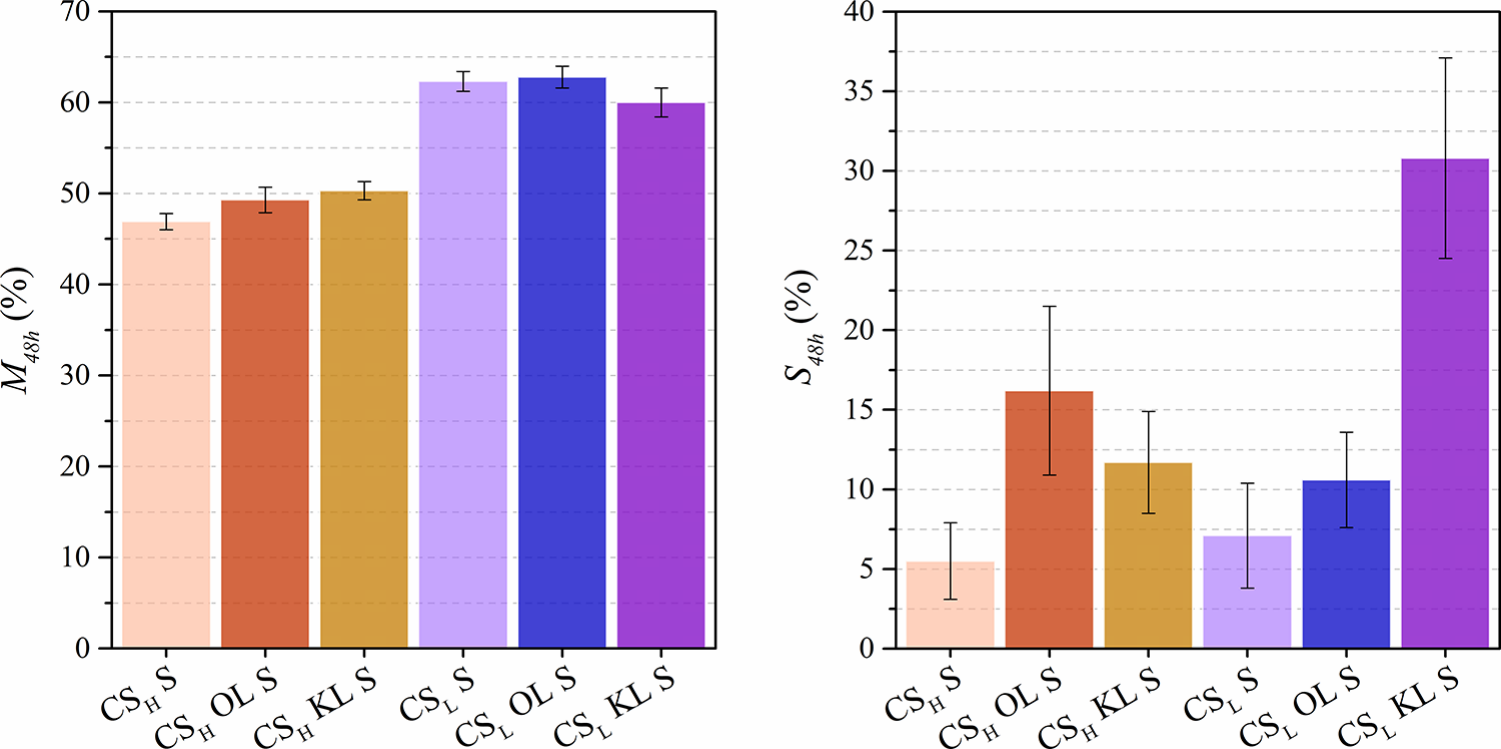
Water uptake (*M*_48h_) and dimensional swelling (*S*_48h_) of the protonated chitosan-lignin composite membranes.

The modified CS_H_ composites, containing chitosan with high *M*_*w*_ and high DDA, absorb less water than the CS_L_ composites. Infrared spectra revealed that CS_H_ composites incorporate larger amounts of sulfate ions. When immersed in the sulfuric acid solution, the negatively charged sulfate ions (SO_4_^2–^) can form Coulombic interactions with the protonated amino groups of chitosan, effectively acting as bifunctional ionic cross-linking agents.^13^ These ionic interactions between chitosan’s amino groups and sulfate ions lead to a dense polymer structure, which could hinder the penetration of water molecules and result in a low water uptake.

The addition of organosolv lignin (OL) and kraft lignin (KL) does not significantly affect the water uptake of the composites but increases the dimensional swelling after water immersion. Lignin interacts with chitosan′s functional groups, reducing the formation of ionic bonds with sulfate ions upon modification. This results in greater dimensional changes, especially in CS_L_ KL S, which incorporates the lowest amount of sulfate groups due to its compact structure.

### 3.3 Calorimetric Phase Transition of the Composite Membranes

Differential scanning calorimetry (DSC) was used to investigate the thermal phase transitions of the protonated chitosan-lignin composites. Figure S3 and Table S6 present the calorimetric thermograms from the first and second heating scans, along with the peak temperatures (*T*) and enthalpies (Δ*h*) of the thermal events. In the first scan, a broad endothermic peak (*T*_1_) around 100 °C corresponds to the evaporation of water,^49–52^ while a shoulder around 120 °C may indicate the evaporation of remnant acetic acid from the sample preparation. In the second scan, an exothermic peak (*T*_2_) around 160 °C followed by an endothermic peak (*T*_3_) around 170 °C reflects the sulfate-induced reduction reaction of chitosan to carbon. The black appearance of the sulfuric acid-treated samples after DSC measurements supports the formation of carbon traces.

In the first heating scan, the protonated CS_H_ composites, based on chitosan with higher molecular mass and higher deacetylation degree (DDA), show greater enthalpies for water evaporation (Δ*h*_1_) than the CS_L_ composites due to their higher hydrophilicity and greater water uptake. Additionally, their water evaporation peak (*T*_1_) occurs at slightly lower temperatures. In the second heating scan, CS_H_ composites exhibit higher enthalpies and peak temperatures for both the exothermic (*T*_2_, Δ*h*_2_) and endothermic (*T*_3_, Δ*h*_3_) events of the reduction reaction, consistent with the increase in sulfate group content, enhancing carbon formation during calorimetric studies.

The incorporation of lignin, particularly organosolv lignin (OL), reduces the enthalpy of water evaporation (Δ*h*_1_). Lignin is a relatively nonpolar molecule, which reduces the hydrophilicity of the composites, consistent with the infrared spectra. Water evaporation (*T*_1_) in the composites containing KL occurs at slightly higher temperatures than in those with OL. Similarly, the reduction reaction (*T*_2_, *T*_3_) in KL-containing composites takes place at slightly higher temperatures due to increased interaction between KL and the chitosan chains.

### 3.4 Relaxation Spectra of the Composite Membranes

#### 3.4.1 Phenomenological Analysis of the Dielectric Spectra

First, the overall dielectric response of the different chitosan-lignin composites is investigated. Figure 3 shows three-dimensional plots of the imaginary permittivity (*ε″*) of the biocomposite membranes after modification by immersion in the 1.0 M sulfuric acid solution.

**Figure 3:**
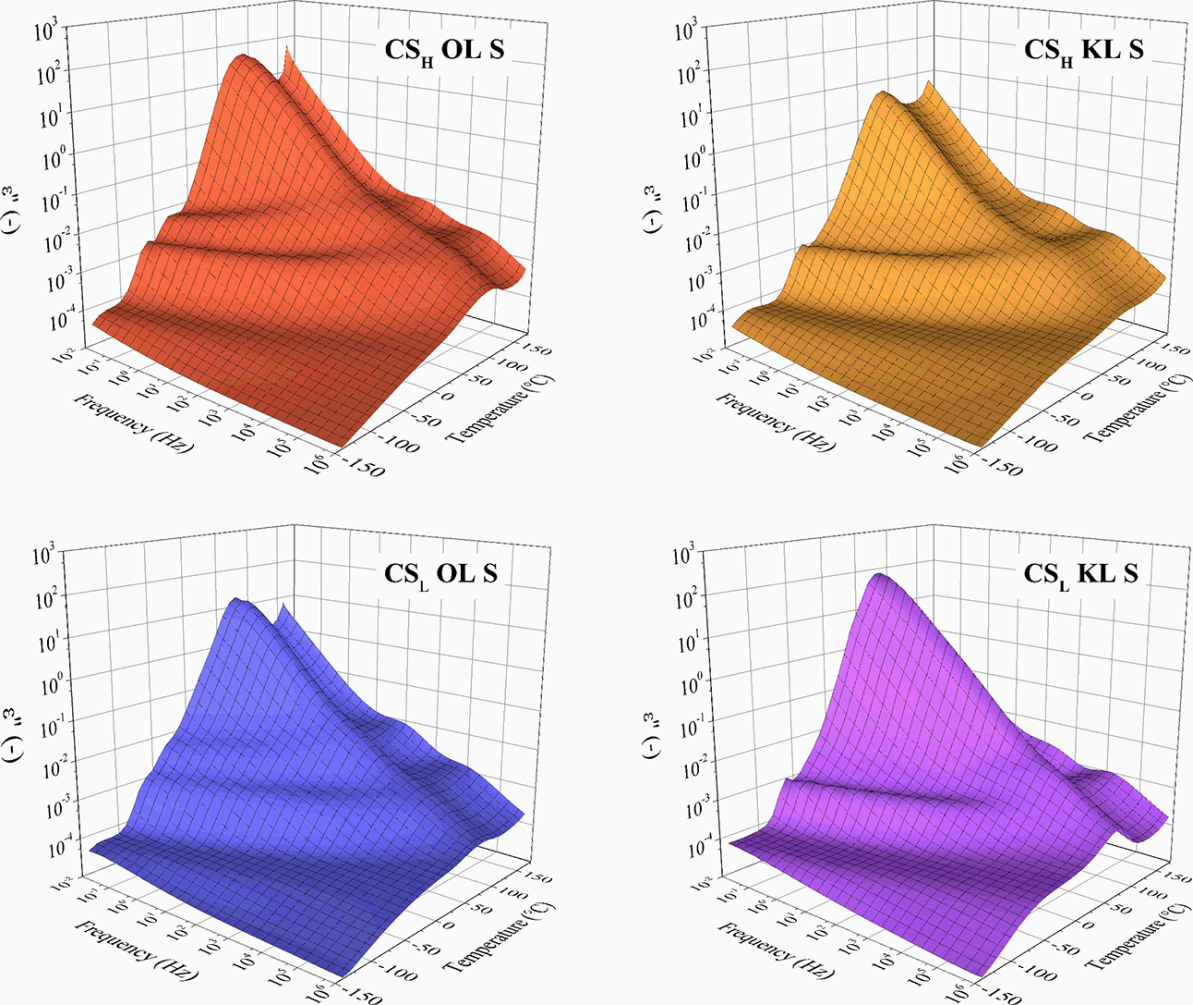
Three-dimensional plot of the imaginary permittivity (*ε″*) of the protonated chitosan-lignin composites.

The two protonated chitosan composites containing organosolv lignin (OL) as a filler show a comparable ε*″*-spectrum, as do the two composites containing kraft lignin (KL). In addition, slight differences in the dielectric relaxation mechanisms are observed for the composites with different types of chitosan as matrix material.

To illustrate the molecular relaxation mechanisms of the sulfuric acid-modified chitosan biocomposites, the dielectric loss factor (*tan­(δ)*) is plotted in isochronal plots as a function of temperature in Figure 4.

**Figure 4:**
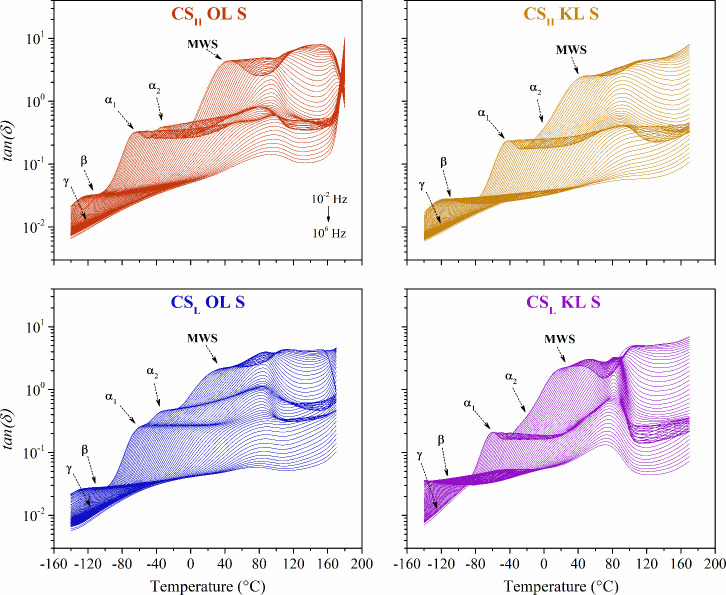
Isochronal plot of the dielectric loss factor *tan­(δ)* of the protonated chitosan-lignin composites.

The chitosan-lignin composites show a complex dielectric spectrum containing the same five dielectric phenomena regardless of the different types of chitosan or lignin. The four molecular relaxations are labeled γ-relaxation, β-relaxation, α_1_-relaxation, and α_2_-relaxation, in order of increasing temperature. The γ-relaxation at low temperatures is attributed to the motions of the incorporated sulfate groups. The β-relaxation results from segmental motions involving the fluctuations of the β-1,4-glycosidic bonds in chitosan's polysaccharide structure.^53^ The α_1_-relaxation and α_2_-relaxation values represent the glass transitions of chitosan and lignin, respectively. The conductivity contribution is designated to an interfacial Maxwell–Wagner–Sillars (MWS) polarization, due to the accumulation of charges at internal boundaries between regions with different conductivity.^54,55^

#### 3.4.2 Cooperativity of the Dielectric Relaxations

The cooperativity of the dielectric molecular dynamics of the protonated chitosan-lignin composites is studied according to the Eyring–Starkweather model (eq 5).^23^ Figure 5 illustrates the activation energy (*E*_*a*_) of the relaxation mechanisms in an Eyring plot, assuming a linear relation between the relaxation time and reciprocal temperature.

**Figure 5:**
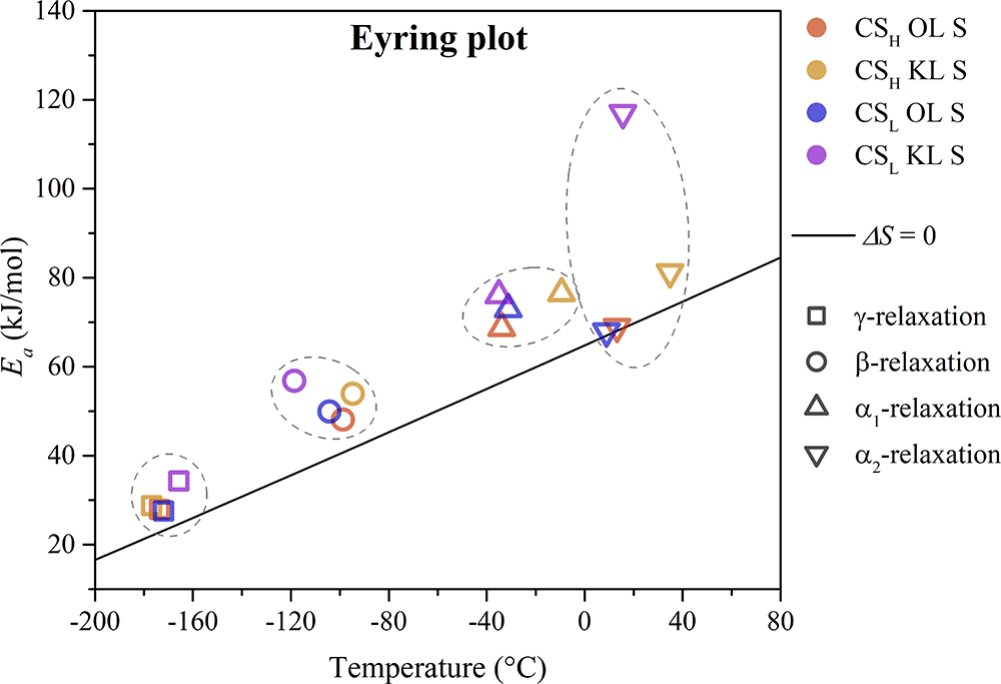
Eyring plot of the molecular relaxation mechanisms of the protonated chitosan-lignin biocomposite membranes at 1 Hz.

The local γ-relaxation and β-relaxation of the chitosan-lignin composites show a slight deviation from the zero-entropy line in the Eyring plot. In general, local motions are not influenced by neighboring units and thus show no cooperativity.^56^ However, the chitosan chains can bind to the functional groups of lignin through secondary intermolecular interactions, hindering their motion.^48^

All of the biocomposites show a clear entropy contribution for the segmental α_1_-relaxation, representing the glass–rubber transition of chitosan. However, there are differences between the composites in the cooperativity of the α_2_-relaxation, associated with lignin’s glass transition. Only the composites containing kraft lignin show a significant deviation from the zero-entropy line, especially for CS_L_ KL S. Typically, relaxations representing glass transitions show a cooperative contribution, due to the connectivity of the chain and the steric hindrance from neighboring segments.^57^ Nevertheless, the segmental motion of the organosolv lignin filler does not seem to be significantly affected.

#### 3.4.3 Analysis of the Local γ-Relaxation and β-Relaxation

The local γ-relaxation and β-relaxation of the sulfuric acid-modified chitosan-lignin composites were found between −140 and 0 °C. Figure 6 plots the dissipation factor *tan­(δ)* of these membranes in an isochronal plot (A) at a frequency of 1 × 10^4^ Hz and in an isothermal plot (B) at −110 °C.

**Figure 6:**
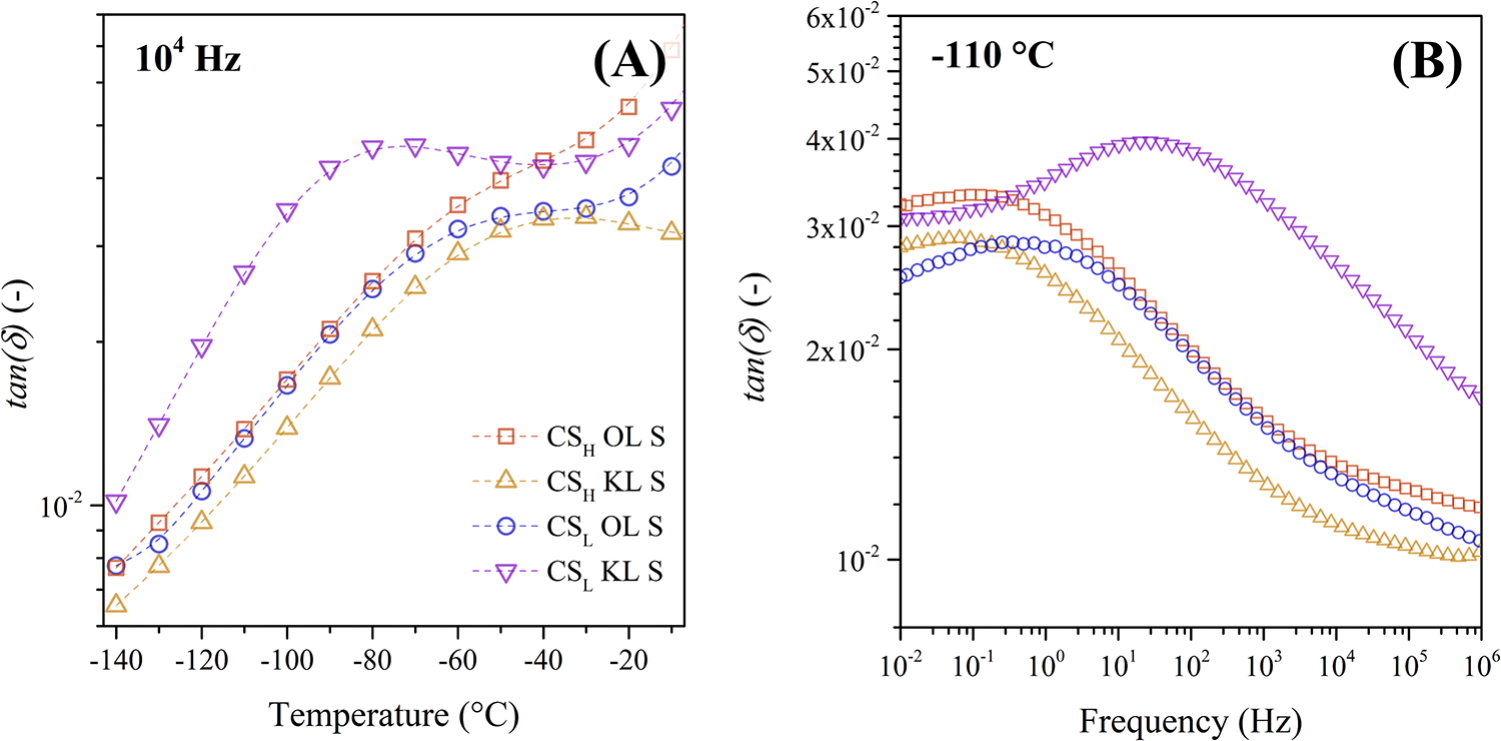
(A) Isochronal plot of the dielectric loss factor (*tan­(δ)*) at 10^4^ Hz and (B) isothermal plot of (tan­(δ)) at −110 °C of the protonated chitosan-lignin composites.

The γ-relaxation has a significantly lower dielectric intensity compared to that of the β-relaxation and appears only as a small shoulder in the isochronal and isothermal plots. The β-relaxation peak of the CS_L_ composites with lower molecular mass (*M*_*w*_) and lower degree of deacetylation (DDA) appears at lower temperatures and higher frequencies, particularly in CS_L_ KL S. In addition, CS_L_ KL S shows a comparably higher peak intensity to the other composites.

Figure 7 represents the relaxation time of the γ-relaxation and β-relaxation as a function of the inverse temperature in an Arrhenius map. Linear temperature dependencies were found in the entire analyzed temperature range for both relaxation and were therefore adjusted to the Arrhenius eq (eq 3). The fit parameters of the γ-relaxation and β-relaxation of the protonated chitosan composites are summarized in Table 2.

**Figure 7:**
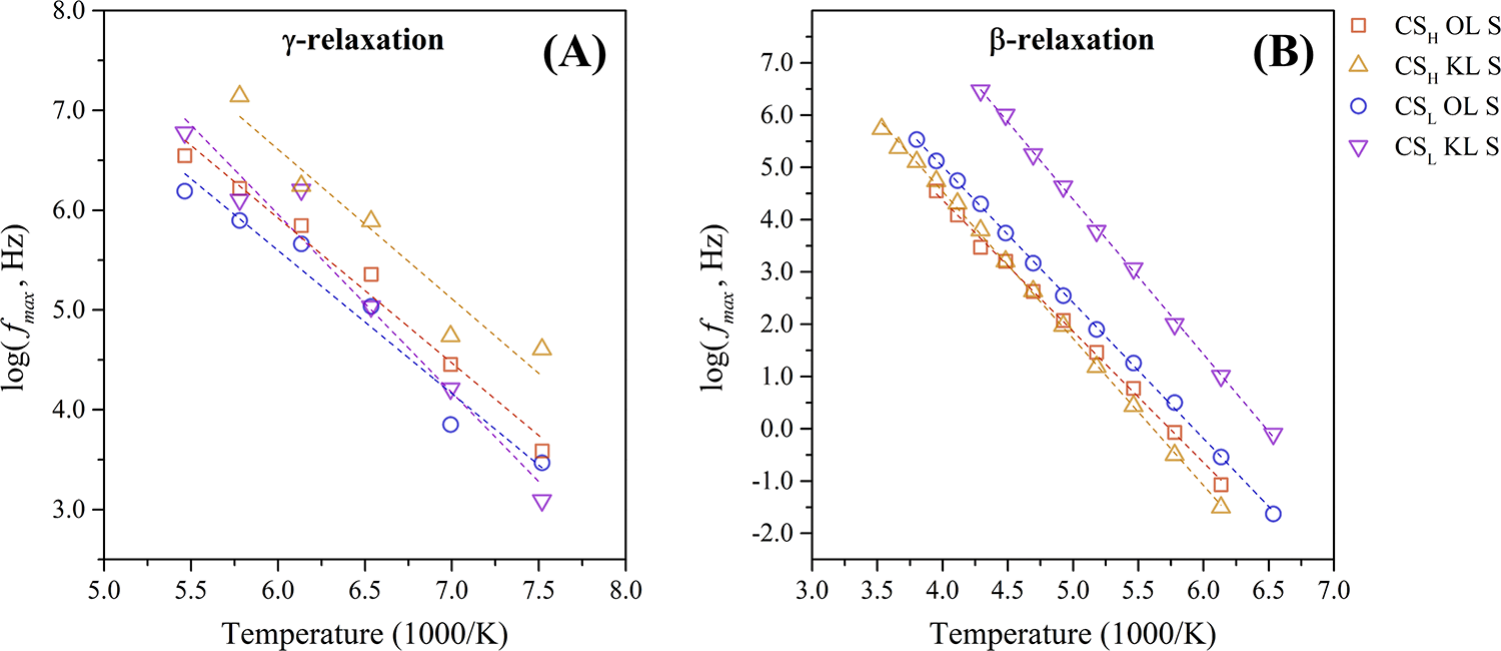
Arrhenius map of (A) γ-relaxation and (B) β-relaxation of the protonated chitosan-lignin composites.

**Table 2:** Arrhenius Fit Parameters of the γ-Relaxation and β-Relaxation of the Protonated Chitosan-Lignin Composites

	**γ-relaxation**		
	*E*_*a*_ (kJ·mol^–1^)	*T*_1 Hz_ (°C)	*R* ^2^
CS_H_ OL S	27.9	–173.6	0.984
CS_H_ KL S	28.7	–176.8	0.935
CS_L_ OL S	27.5	–171.9	0.961
CS_L_ KL S	34.3	–165.7	0.965

	**β-relaxation**		
	*E*_*a*_ (kJ·mol^–1^)	*T*_1 Hz_ (°C)	*R* ^2^
CS_H_ OL S	48.1	–98.7	0.998
CS_H_ KL S	53.9	–94.7	0.999
CS_L_ OL S	49.9	–104.2	0.999
CS_L_ KL S	56.8	–118.6	0.999

The γ-relaxation of the four protonated chitosan-lignin composites shows comparable activation energies (*E*_*a*_) between 27.5 and 34.3 kJ/mol. These low activation energies are typical for local movements involving only a small number of atoms.^58^ According to these findings, the γ-relaxation is assigned to the movement of the sulfate groups incorporated by immersion of the biocomposites in a 1.0 M sulfuric acid solution.

The γ-relaxation of the chitosan composites containing kraft lignin (KL) shows a slightly higher activation energy compared to those containing organosolv lignin (OL). This suggests that the lignin filler directly affects the composite's susceptibility to the modification and incorporation of a sulfate group by immersion in the sulfuric acid solution.

The β-relaxation is attributed to local fluctuations of the β-1,4-glycosidic bonds in the chitosan chain. Einfeldt et al. extensively studied this process for various polysaccharides containing glycosidic links, including chitosan, and reported *E*_*a*_-values around 45 kJ/mol.^53^ In this study, the β-relaxation of the chitosan-lignin composites reveals slightly higher activation energies between 48.1 and 56.8 kJ/mol. This increase is likely due to intermolecular interactions between lignin and chitosan, which impede the movement of the glycosidic bonds and increase the thermal activation and cooperativity of the β-relaxation process.^59^ In addition, incorporated sulfate groups can form ionic bridges between chitosan chains, further restricting the movement.^13^

Composites containing KL exhibit higher *E*_*a*_ values for the β-relaxation mechanism than those with OL. KL has a lower molecular mass (*M*_w,KL_ = 2.5 kDa) than OL (*M*_w,OL_ = 9.5 kDa) (see Table S4), allowing for better distribution within the chitosan matrix. In addition, KL has more phenolic OH groups (*A*_750 nm_ = 1.24) compared to OL (*A*_750 nm_ = 0.43), as shown in Figure S2 and Table S5. The combination of smaller size and a greater number of polar functional groups enables KL to form stronger intermolecular interactions with chitosan chains, increasing the activation energy of the β-relaxation.

In addition, the composites with a chitosan type of high molecular mass and high degree of deacetylation (DDA) (CS_H_) show slightly elevated *E*_*a*_-values for the β-relaxation in comparison with the CS_L_ composites. CS_H_ contains more polar amine groups in the chain, which can form more Coulombic interactions with the sulfate groups, which impedes chitosan’s movement and increases the thermal activation of the β-relaxation. Furthermore, the β-relaxation of the CS_H_ composites occurs at higher temperatures compared to the CS_L_ composites, presumably due to their longer chain length.

#### 3.4.4 Analysis of the α_1_-Relaxation and α_2_-Relaxation

The α-relaxation in dielectric studies is commonly assigned to the glass transitions of polymers. In these chitosan-lignin biocomposites, two separate α-relaxations appear due to the glass transition of both its constituents. The relaxation at lower temperature (α_1_), related to segmental motions of the chitosan chains, was found between −50 and 60 °C for the protonated composites. Figure 8A plots the dielectric loss factor tan­(δ) at a frequency of 1 × 10^2^ Hz over this temperature range.

**Figure 8:**
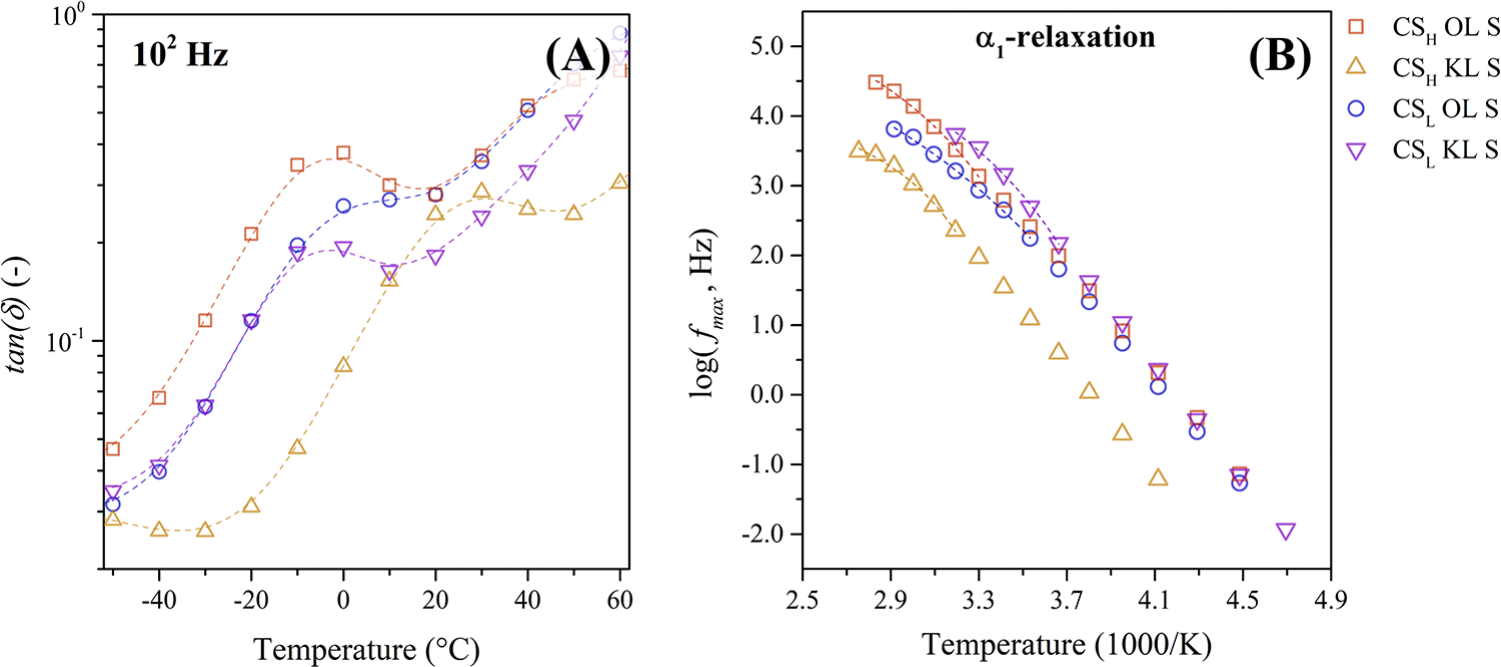
(A) Isochronal plot of the dielectric loss factor (tan­(δ)) at 10^2^ Hz and (B) Arrhenius map of the α_1_-relaxation of the protonated chitosan-lignin composites.

The CS_H_ composites show a higher peak intensity related to the α_1_-relaxation compared to the CS_L_ composites, which could be due to the higher molecular mass resulting in longer polymer chains. In addition, the intensity of the composites containing organosolv lignin shows higher peaks compared to the ones with kraft lignin. Furthermore, the relaxation peak of the different chitosan-lignin composites occurs at different temperatures, with CS_H_ KL S having the highest temperature.

The α_1_-relaxation of the chitosan-lignin composites shows a linear temperature dependence at lower temperatures and a curvature at higher temperatures in the Arrhenius map in Figure 8B. This nonlinear behavior is typical for segmental motions of polymers, confirming the assignment of this process to the glass transition temperature of chitosan. Accordingly, the α_1_-relaxation was adjusted to the VFTH model (eq 4) and the results of the fit are summarized in Table 3.

**Table 3:** VFTH Fit Parameters of the α_1_-Relaxation of the Protonated Chitosan-Lignin Composites

	**α**_**1**_ **-relaxation**			
	*f*_0_ (Hz)	*D*	*T*_V_ (K)	*R* ^2^
CS_H_ OL S	6.51 ± 0.43	2.45 ± 0.97	230.6 ± 14.7	0.997
CS_H_ KL S	4.66 ± 0.36	0.95 ± 0.46	265.9 ± 13.3	0.990
CS_L_ OL S	6.53 ± 0.48	5.58 ± 2.11	180.8 ± 17.4	0.998
CS_L_ KL S	6.27 ± 0.86	2.79 ± 1.73	210.9 ± 19.7	0.994

The sulfuric acid-modified chitosan-lignin composites show strong differences in the Vogel temperature (*T*_V_) with values ranging from 180.8 to 265.9 K. The Vogel temperature (*T*_V_) is often determined to characterize polymer behavior near the glass transition (*T*_V_ < *T*_g_).^58^ The CS_H_ composites show higher *T*_V_-values compared to the CS_L_ composites, as they consist of longer polymer chains and thus require more thermal energy to induce the segmental motion, according to the Flory–Fox equation, due to less free volume and restricted chain mobility.^60^ In addition, the CS_H_ composites contain comparatively more amino groups that can form strong intermolecular interactions via hydrogen bonding, which further restricts segmental mobility.

Furthermore, the composites with KL show slightly higher temperatures compared to those containing OL, due to previously mentioned interactions of lignin with the chitosan chains, which restrict the movements of the polymer segments. Kraft lignin can distribute itself better in the matrix due to its small molecular size and thus can create strong intermolecular interactions.

On the other hand, the fragility parameter (*D*) of the protonated biocomposites behaves contrary to the Vogel temperature, where membranes with high *T*_V_ values show low *D* values. Polymers with low *D*-values are generally regarded as fragile glass formers, exhibiting abrupt changes as they approach the glass–rubber transition.^61^ The CS_H_ composites with longer polymer chains are more fragile due to more possible entanglements, making the material less flexible. In addition, the interactions with lignin restrict the movement of the chains, which increases the fragility, especially kraft lignin.

Next, the α_2_-relaxation of the chitosan-lignin composites after immersion in a 1.0 M sulfuric acid solution is investigated, which is associated with the glass transition of the different lignin fillers. Figure 9A plots the dielectric loss factor *tan­(δ)* at 10 ° Hz between −20 and 60 °C.

**Figure 9:**
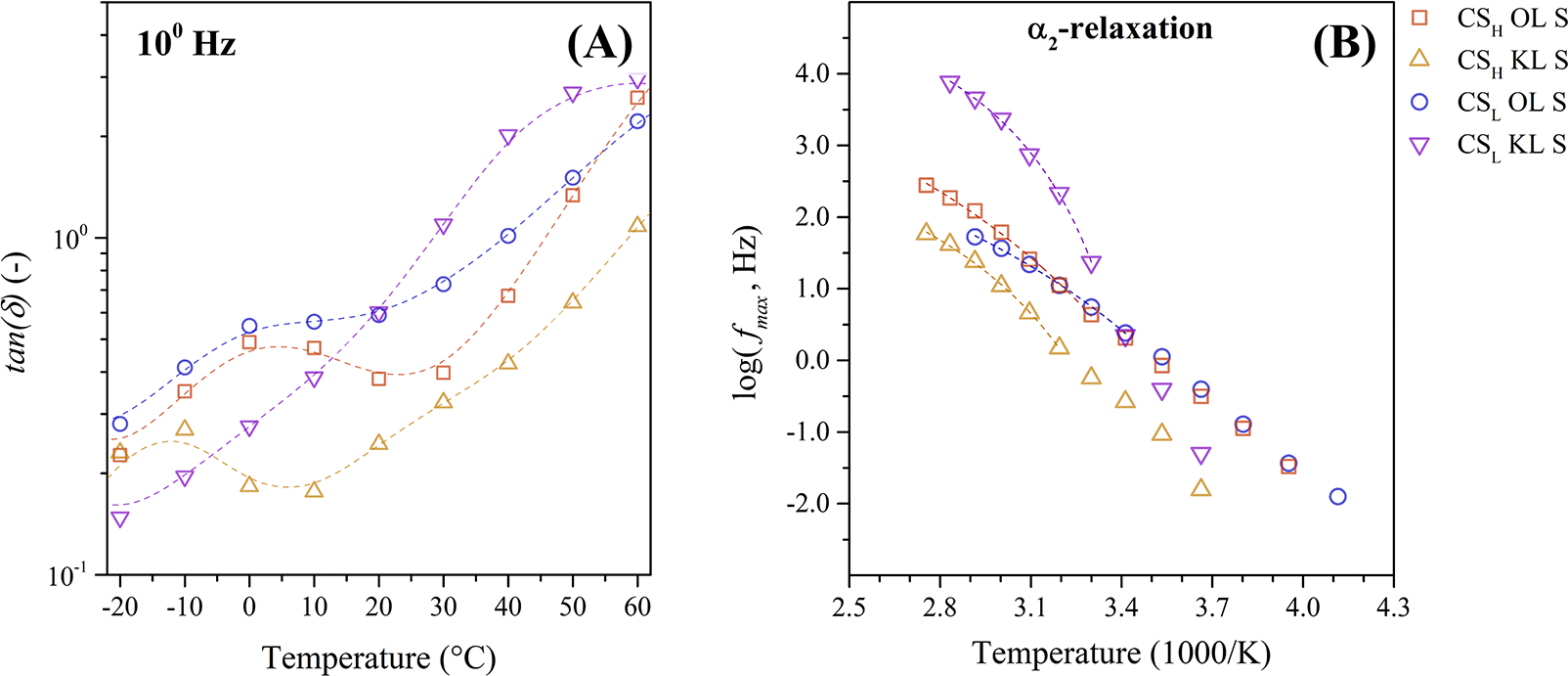
(A) Isochronal plot of the dielectric loss factor (*tan­(δ)*) at 10 ° Hz and (B) Arrhenius map of the α_2_-relaxation of the protonated chitosan-lignin composites.

The α_2_-relaxation peak in the isochronal *tan­(δ)*-plot overlaps with the conductivity contribution from MWS polarization at higher temperatures. The composites containing kraft lignin (KL) show significantly lower peak intensity compared with those with organosolv lignin, with the α_2_-peak appearing as a shoulder in the KL composites. Additionally, the α_2_-relaxation peak of the CS_L_ composites exhibits a slightly higher intensity compared to the CS_H_ composites.

Figure 9B shows the Arrhenius map of the α_2_-relaxation of the sulfuric acid-modified chitosan-lignin composites, in which they all demonstrate a nonlinear temperature dependence. Accordingly, they were adjusted to the VFTH equation (eq 4) with the results of the fit summarized in Table 4.

**Table 4:** VFTH Fit Parameters of the α_2_-Relaxation of the Protonated Chitosan-Lignin Composites

	**α**_**2**_ **-relaxation**			
	*f*_0_ (Hz)	*D*	*T*_V_ (K)	*R* ^2^
CS_H_ OL S	5.42 ± 0.66	5.08 ± 2.27	207.6 ± 20.2	0.997
CS_H_ KL S	3.76 ± 0.31	1.98 ± 0.51	252.5 ± 8.5	0.998
CS_L_ OL S	4.35 ± 0.45	4.46 ± 1.59	197.1 ± 15.8	0.999
CS_L_ KL S	5.75 ± 0.21	1.39 ± 0.21	266.3 ± 3.4	0.999

The α_2_-relaxation of the protonated composites with organosolv lignin exhibits a Vogel temperature (*T*_*V*_) of 197.1 and 207.6 °C, while the kraft lignin composites show *T*_V_-values of 252.5 and 266.3 °C. The significant differences between the composites with different lignin types prove the assignment of the α_2_-relaxation to the glass transition of the lignin filler. Furthermore, the KL molecules interact more strongly with the chitosan chains, as previously demonstrated. These strong interactions increase the thermal energy required to initiate lignin’s segmental motion. In addition, the sulfonated composites containing KL show significantly lower fragility parameters (*D*) compared to those with OL, due to its increased interaction with the chitosan chains.

#### 3.4.5 Electron Conductivity

Next, the electron conductivity of the dry chitosan-lignin composite membranes after modification by immersion in a 1.0 M sulfuric acid solution is investigated. The isothermal plot of the real part of the conductivity (*σ*′) between 10^–2^ and 10^5^ Hz can be found in Figure S4 in the Supporting Information.

At low frequencies, the protonated composites show a plateau around 0 °C that moves to higher frequencies as the temperature rises. In the high-frequency zone, the biocomposites show a linear increase with some bulges, which represent molecular relaxations that affect the charge transfer mechanisms. The low-frequency plateau represents the bulk conductivity of the materials and was adjusted by applying Jonscher’s power law (eq 6). The obtained bulk electron conductivity (*σ*_0_) is plotted as a function of the inverse temperature in Figure 10.

**Figure 10:**
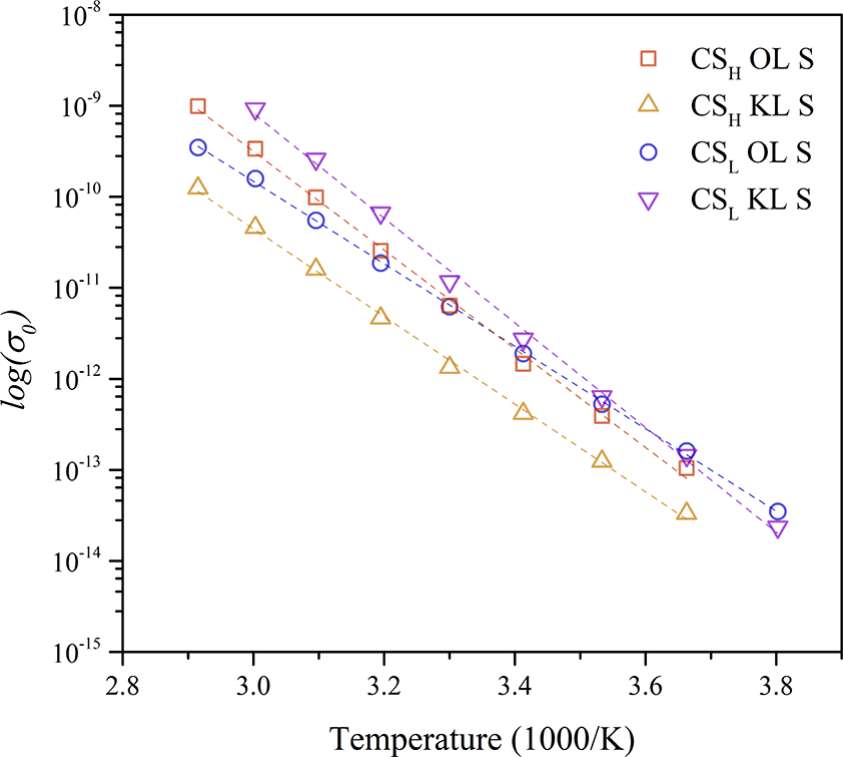
Arrhenius map of the bulk conductivity (*σ*_0_) of the protonated chitosan-lignin composite membranes.

Overall, the chitosan-lignin composites show electron conductivities between 10^–14^ to 10^–9^ S/cm between −10 and 80 °C. According to these results, the composites can be classified as electrical insulators, which is an important characteristic in fuel cell applications.

The bulk conductivity (*σ*_0_) of the chitosan-lignin membranes shows a linear temperature dependence in the Arrhenius map in Figure 10. This linearity indicates that charge transport within the sulfonated biocomposites primarily occurs through the vehicular mechanism, in which the protons diffuse as hydrated proton species.^62,63^

The linear temperature dependence of the bulk conductivity (*σ*_0_) was adjusted to the Arrhenius model to study its thermal activation. Table 5 summarizes the parameters of the linear fit and the activation energy of the electron conductivity process of the sulfuric acid-modified chitosan-lignin composite membranes. The composites show *E*_*a*_-values between 86.8 and 110.2 kJ/mol, in an increasing order: CS_L_ KL S > CS_H_ OL S > CS_H_ KL S > CS_L_ OL S. These values are typical for chitosan-based materials.^13,59^

**Table 5:** Activation Energy from the Arrhenius Fit of the Electric Bulk Conductivity of the Protonated Chitosan-Lignin Composite Membranes

	intercept	slope	*R* ^2^	*E*_*a*_ (kJ·mol^–1^)
CS_H_ OL S	6.77	–5.43	0.998	103.9
CS_H_ KL S	4.10	–4.82	0.999	92.3
CS_L_ OL S	3.77	–4.54	0.999	86.8
CS_L_ KL S	8.18	–5.76	0.998	110.2

#### 3.4.6 Proton Conductivity under Hydrated Conditions

Furthermore, the proton conductivity of the chitosan-lignin membranes after protonation in a 1.0 M sulfuric acid solution under hydrated conditions was investigated by dielectric impedance spectroscopy. Figure S5 illustrates the modulus of the impedance (|*Z*|) and the phase angle (Φ) of the biobased composites at 60 °C as a function of frequency in a Bode plot. The high-frequency peak is due to the bulk resistance (*R*_0_) and was used for the calculation of the proton conductivity (*σ*_Prot_), while the low-frequency curvatures are assigned to molecular relaxation processes.

The calculated proton conductivity of the protonated biocomposite membranes at 60 °C is shown in Figure 11.

**Figure 11:**
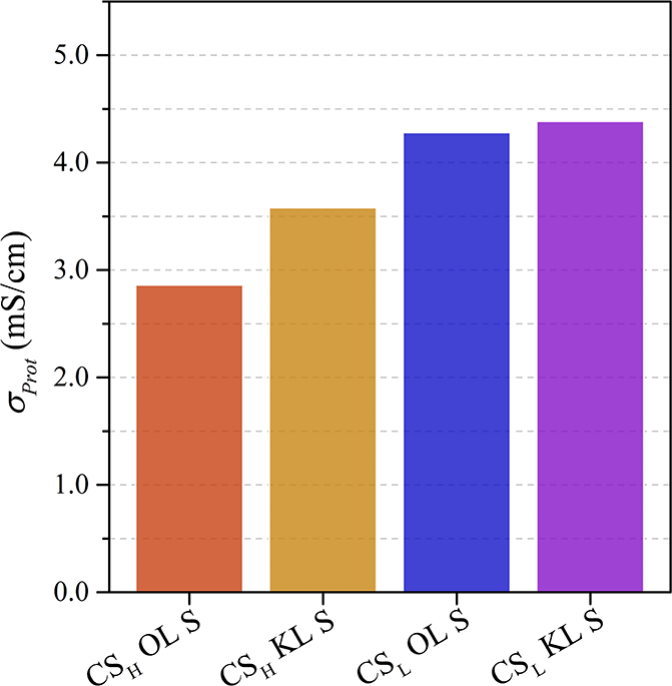
Proton conductivity (*σ*_prot_) of the protonated composite membranes at 60 °C under hydrated conditions.

The chitosan-lignin biocomposites exhibit proton conductivities between 2.86 × 10^–3^ and 4.38 × 10^–3^ S/cm at 60 °C, comparable to values reported for other modified biopolymers.^64,65^ However, these conductivities remain lower than that of commercial Nafion (∼100 × 10^–3^ S/cm), indicating the need for further optimization. Nonetheless, the biocomposites offer a significant environmental and economic advantage, as they can be derived from industrial waste products, reducing reliance on petroleum-based resources and promoting the principles of a circular economy.

The CS_L_ composites prepared from low molecular mass chitosan with a low degree of deacetylation show higher proton conductivities compared to the CS_H_ composites. In the water uptake studies, the CS_L_ composites demonstrated higher water absorption, which accounts for these results. High water uptake enhances the vehicular charge transfer mechanism, in which protons diffuse through the membrane as hydrated species, leading to elevated proton conductivities.^50^ In addition, the composites containing kraft lignin as a filler show slightly higher proton conductivities than those with organosolv lignin.

## 4 Conclusions

This study highlights the potential of biobased chitosan-lignin composites as promising materials for energy-related applications, such as fuel cells. By incorporating two types of chitosan with different molecular masses and degrees of deacetylation (DDA), along with kraft or organosolv lignin as filler, the structural, thermal, water absorption, dielectric, and conductive properties of the resulting membranes were altered.

Chitosan composites with high molecular mass and high DDA (CS_H_) contain more amino groups and thus exhibit increased hydrophilicity and enhanced sulfate group incorporation than those with low molecular mass and low DDA (CS_L_). The increased ionic interactions of the CS_H_ composites with the sulfate groups result in a dense polymer structure, hindering water absorption.

Lignin incorporation reduces the hydrophilicity and increases the dimensional swelling of the composites when immersed in water. Kraft lignin (KL) induces more significant structural changes due to its smaller size and higher phenolic content, which facilitates its even distribution and interaction with chitosan’s amino groups.

Dielectric analysis identified four relaxation mechanisms (γ-, β-, α_1_-, and α_2_-relaxation) and one interfacial Maxwell–Wagner-Sillars polarization. The β-glycosidic bond mobility in CS_H_ composites is more restricted due to more ionic interactions with sulfate groups. Lignin addition increases the temperature of the segmental α_1_-relaxation, particularly when KL is used, due to its strong intermolecular interactions. The longer polymer chains of the CS_H_ composites are less flexible, leading to increased fragility.

Electron conductivity measurements show that the biobased composite membranes are electrical insulators with values ranging from 10^–15^ to 10^–8^ S/cm between −10 and 170 °C. Under hydrated conditions, the composites exhibit proton conductivities between 2.9 and 4.4 mS/cm, with CS_L_ composites demonstrating improved proton transport due to higher water uptake.

The versatility of these composites underscores their potential as materials in energy-related applications such as fuel cells.

## Supplementary Material



## Notes

Experimental details and characterization of organosolv lignin (OL) and kraft lignin (KL) fillers; DSC thermograms, electron conductivity plots, and Bode plots of protonated chitosan-lignin composites (PDF)
