# Supplementary material for: Dielectric Characterization of Protonated Chitosan-Lignin Biocomposite Membranes: Influence of Chitosan and Lignin Types
Source: Biomacromolecules. 2025 Aug 26;26(9):6140–51. doi: 10.1021/acs.biomac.5c01090 (PMC12421681; doi:10.1021/acs.biomac.5c01090)
Supplement: Supplementary file 1 [file bm5c01090_si_001.pdf]

Supplementary material for: **Dielectric Characterization of Protonated Chitosan-Lignin Biocomposite Membranes: Influence of Chitosan and Lignin Types**

M.H. Wolf[1], N. Izaguirre[2], J. Labidi[2], A. Ribes-Greus[1,*]

[1]Research Institute for Materials Technology, Universitat Politècnica de València, 46022 Valencia, Spain.

[2]Chemical and Environmental Engineering Department, Universidad del País Vasco, 20018 Donostia, Spain

*Corresponding authors: aribes@ter.upv.es

1. Physico-chemical characterization of lignin types

1.1. Fourier-transform infrared (FTIR) spectroscopy

The acquisition of the FTIR spectra of the lignin types were performed analogous to the chitosan-lignin composite membranes with a Nicolet iS50 FTIR Spectrometer.

1.2. Pyrolysis-Gas chromatography-Mass spectroscopy (Py-GC–MS) analysis

The gases from pyrolysis of the lignin types were analyzed with a gas chromatograph coupled to a mass spectrometer type 6890 GC/5973 Network MSD from Agilent Technologies (Santa Clara, CA, USA). Pyrolysis was performed with a 5150 Pyroprobe filament pyrolyser from CDS Analytical (Oxford, PA, USA). The lignin types (400-800 mg) were heated in a quartz crucible at a rate of 20 °C/ms until reaching 600 ºC and maintained for 15 seconds. Then, the pyrolysates under inert conditions were purged into the fused-silica capillary GC column Equity-1701 (30 m × 0.20 mm × 0.25 µm). The GC oven program started at 50 °C and was held for 2 min. Then it was raised to 120 °C at 10 °C/min and was held for 5 min, after that it was raised to 280 °C at 10 °C/min, held for 8 min and finally raised to 300 °C at 10 °C/min and was held for 10 min. The compounds were identified by comparing their mass spectra with the National Institute of Standards Library (NIST) and with compounds reported in the literature.

1.3. Gel permeation chromatography (GPC)

The molecular weight distribution of the lignin types was determined with a JASCO LC-Net II/ADC equipment (Tokyo, Japan). The device is equipped with a PolarGel-M column (300 mm × 7.5 mm), PolarGel-M guard (50 mm × 7.5 mm), and the RI-2031 Plus Intelligent refractive index detector. The samples were dissolved in a dimethylformamide solution (50 ppm) containing 1.0 g/L lithium bromide

which was also used as the mobile phase of the column. A column temperature of 40 ºC and a flow rate of 0.7 mL/min was chosen. Polystyrene standards were used for the calibration curve.

1.4. UV-VIS Spectroscopy

For the quantitative determination of the total phenolic OH groups, the spectrophotometric Folin-Ciocalteau method was used. The lignin types were dissolved in dimethyl sulfoxide (c = 2 g/L). To 0.5 mL of the lignin solution 2.5 mL of Folin-Ciocalteau reactive and 5 mL of Na_2Co_3 solution (c = 200 g/L) were added. Next distilled water was added to the solutions to reach a total volume of 50 mL and then they were sonicated at 40 °C for 30 min. The absorbance of the solutions at 750 nm was determined in triplicates with a Jasco V-630 spectrophotometer.

For the qualitative analysis, 5 mg of each lignin type was dissolved in dioxane/H_2O (95/5, v/v). This solution was further diluted (1 mL in 9 mL dioxane/H_2O (50/50, v/v)) and then measured in the UV/VIS spectrophotometer between 400 and 260 nm.

2. Characterization of the lignin types

2.1. Chemical structure of the lignin types

The chemical structure and functional groups of the lignin types can be ascertained from the infrared spectra between 3800 cm^{-1} and 800 cm^{-1} in **Figure S1**. The most relevant absorption bands and their designation are listed in **Table S1**.

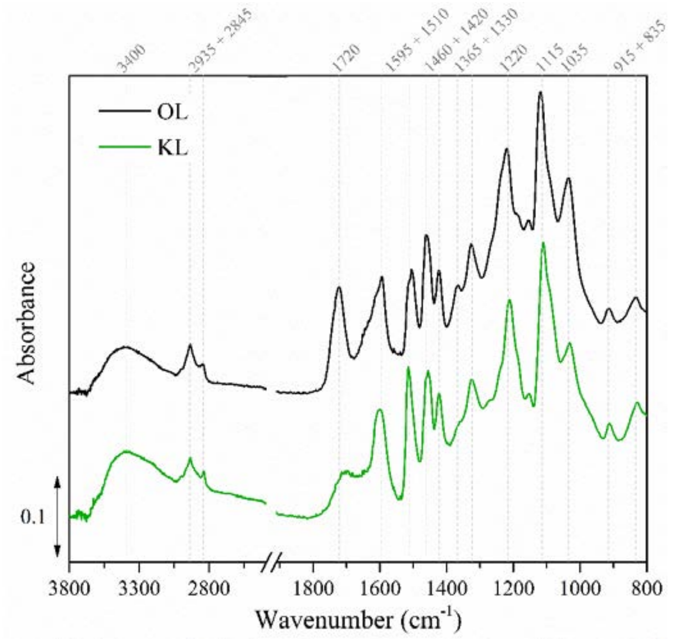

Figure S1: FTIR spectra of organosolv lignin (OL) and kraft lignin (KL)

The two different lignin types show differences in their chemical structure and functional groups, even though they are both extracted from Eucalyptus. The different extraction methods result in structural changes that can be ascertained in the infrared spectra. Kraft lignin (KL) was extracted from eucalyptus kraft pulp while organosolv lignin (OL) was extracted from eucalyptus chips with a formic acid and acetic acid treatment.

Table S1: Infrared bands of lignin and their designation

Wavenumber (cm^{-1})	Designation
3400	O-H stretching
2935 + 2845	C-H stretching in CH_2 + CH_3
1720	C=O stretching unconjugated
1595 + 1510 + 1420	Aromatic skeleton vibrations
1460	C-H deformations
1365	Aliphatic C-H stretching in CH_3
1330	Syringyl ring breathing with C-O stretching
1220	C-C + C-O stretching
1115	Aromatic C-H in-plane deformation of syringyl unit
1035	Aromatic C-H in-plane deformation of guaiacyl unit
915 + 835	Aromatic C-H out of plane deformation

Kraft lignin (KL) shows higher absorbance at the broad band of the OH groups at 3400 cm^{-1} compared to the organosolv lignin (OL). The kraft delignification process employs hard conditions in comparison to the organosolv acetic process, which leads to the breaking of more lignin bonds and the creation of small and functionalized lignin.

Furthermore, OL shows higher absorbance at the bands at 1220, 1115, and 1035 cm^{-1}, associated to the C-C and C-O stretching as well as the aromatic C-H deformations of the lignin units, respectively. Organosolv lignin has a higher molecular weight compared to the kraft lignin and therefore has comparatively less functional groups located on the outside and more phenylpropane backbone. These findings suggest that OL has a more condensed structure compared to KL. This condensed structure of OL could lead to fewer accessible functional groups within the tightly packed lignin network. This can also be appreciated from the higher absorbance found for organosolv lignin for the C-H stretching of the methoxy groups ($O-CH_3$) at 1365 cm^{-1} in comparison with kraft lignin.

2.2. Composition of the lignin types by Py-GC-MS analysis

Py-GC-MS analysis was performed on the different lignin types to determine the S, G, and H unit content by examining the gaseous pyrolysis products after separation in a gas chromatograph using mass spectroscopy. **Table S2** summarizes the results of the analyzed mass spectroscopy areas and **Table S3** lists the resulting chemical composition of the different lignin types.

Table S2: Py-GC-MS compound list for organosolv lignin (OL) and kraft lignin (KL)

Lignin unit	Compound	m/z	Area	
			**OL**	**KL**
H	o-Cresol	96/95/39	0.27	0.46
	2, 4-Xiletol	122/107/121	0.43	0.94
	p-Cresol	107/108/77/79	0.71	0.89
	Phenol	94/66/65	0.54	0.50
	4-Ethylphenol	107/122/77	0.53	-
G	3-Methoxycatechol	140/125/97	5.33	7.08
	Guaiacol	109/124/81	3.44	3.71
	4-Methylguaiacol	138/123/95	3.15	3.38
	4-Ethylguaiacol	137/152/15	1.62	2.66
	4-Vinylguaiacol	135/150/107	2.31	2.38
	3-Methylguaiacol	123/138/77	0.51	0.99
	Isoeugenol	164/77/149	1.82	0.58
	4-Propylguaiacol	137/166/122	0.89	0.92
	4-Methylcatechol	124/123/78	0.18	0.24
	3-Methoxy-5-methylphenol	138/109/107	-	0.37
	Acetoguaiacone	151/166/123	0.28	0.85
	Guaiacyl acetone	137/180/122	0.23	0.35
	Vanillin	151/152/81	0.55	-
	Catechol	110/92/64	0.42	0.18
S	Syringol	154/139/111	11.04	19.84
	4-Methylsyringol	168/153/125	11.39	13.25
	4-Ethylsyringol	167/182/168/77	3.90	4.32
	4-Vinylsyringol	180/165/137	3.34	4.62
	Acetosyringone	181/196/43	4.62	1.10
	4-Allylsyringol	194/91/119	4.19	3.07
	Syringaldehyde	182/181/111	1.94	0.49
	Propiosyringone	181/182/210	1.17	0.37
	4-Propylsyringol	167/196/168	0.50	1.25

Table S3: Pyrolysis analysis of organosolv lignin (OL) and kraft lignin (KL).

Lignin	H unit (%)	G unit (%)	S unit (%)	Analyzed area (%)
**OL**	3.80	31.73	64.47	65.3
**KL**	3.73	31.67	64.60	74.8

The Py-GC-MS analysis shows that both lignin types have the same chemical composition, as both are extracted from eucalyptus sources. This is in accordance with the infrared spectra, where the different lignin types show comparable absorbances at the bands representative of the different lignin units.

2.3. Molecular weight of lignin types

The molecular weight of the two lignin types was determined by GPC analysis with the results being summarized in **Table S4**.

Table S4: Number average molecular weight (M_n), weight average molecular weight (M_w), and polydispersity index (*PDI*) of organosolv lignin (OL) and kraft lignin (KL).

Lignin	M_n (Da)	M_w (Da)	*PDI*
**OL**	2303	9528	4.14
**KL**	810	2541	3.13

Kraft lignin (KL) from eucalyptus black liquor has approximately four times lower molecular weight compared lignin extracted from eucalyptus by organosolv acid extraction (OL), due to the harsher process conditions which leads to the breaking of lignin bonds. In addition, the polydispersity index (PDI) of organosolv lignin is slightly higher.

2.4. UV absorbance and OH group content

UV/VIS spectroscopy was used to quantify the phenolic OH group content of the two different lignin types by using the Folin-Ciocalteau method. **Table S5** shows the absorbance at 750 nm of organosolv lignin (OL) and kraft lignin (KL).

It can be seen that kraft lignin (KL) has a higher reductive phenolic content, e.g. phenolic OH groups, compared to organosolv lignin (OL). This is in accordance with the FTIR results, where KL showed higher absorption at the band representing the OH stretching vibration.

Table S5: UV/VIS absorbance at 750 nm through Folin-Cioiclteau method of organosolv lignin (OL) and kraft lignin (KL).

Lignin	A_{750nm}
**OL**	0.434 ± 0.03
**KL**	1.239 ± 0.01

Furthermore, UV/VIS spectroscopy was used to qualitatively assess the phenolic OH conjugation in the lignin types. **Figure S2** illustrates the UV absorption between 260 and 400 nm of organosolv lignin (OL) and kraft lignin (KL).

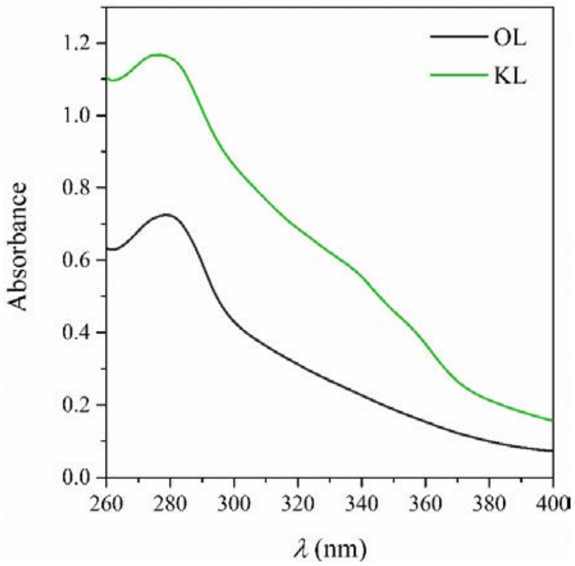

Figure S2: UV absorbance between 260 and 400 nm of the lignin types in a dioxane/H_2O solution

Kraft lignin shows comparably higher absorbance in the measured UV range, due to a higher amount of phenolic OH groups. This is in accordance with the found infrared results and spectrophotometric Folin-Ciocalteau results. Moreover, KL shows relatively less non-conjugated OH groups (280 nm) and relatively more conjugated OH groups (320-380 nm) compared to OL. Generally, the conjugated OH groups are more reactive, due to resonance stabilization and increased acidity.

3. Characterization of the chitosan-lignin biocomposite membranes

3.1. Calorimetric phase transition of the composite membranes

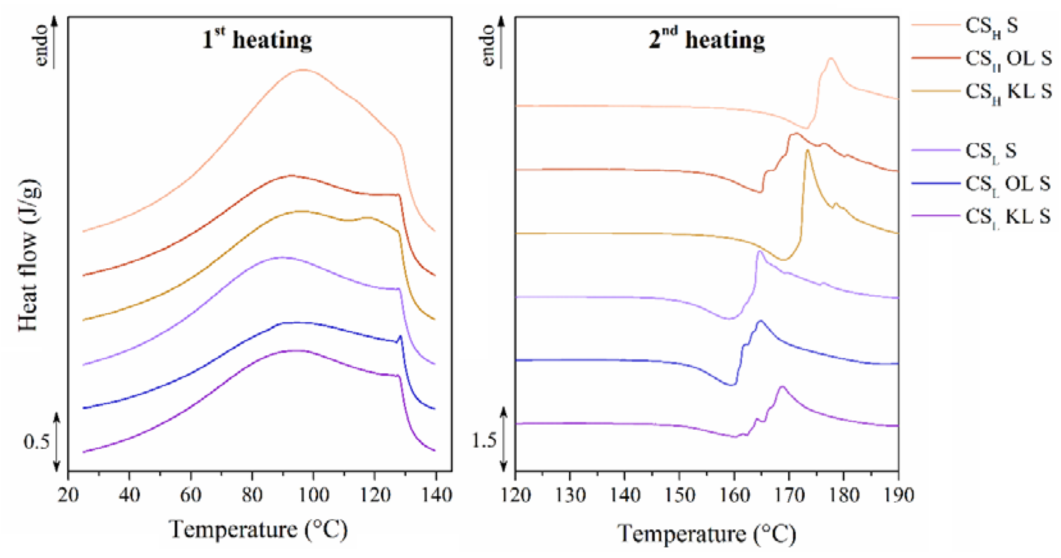

Figure S3: DSC thermograms of the 1st heating scan (top) and the second heating scan (bottom) of the protonated chitosan-lignin composites

Table S6: Calorimetric results of the 1st and 2nd heating scans of the protonated chitosan-lignin composites.

	**1st heating**		**2nd heating**			
	T_1 (ºC)	Δh_1 ($J \cdot g^{-1}$)	T_2 (ºC)	Δh_2 ($J \cdot g^{-1}$)	T_3 (ºC)	Δh_3 ($J \cdot g^{-1}$)
**CS_H S**	96.9 ± 0.2	236 ± 13	172.0 ± 1.1	-37.2 ± 0.1	175.9 ± 1.8	89.5 ± 1.4
**CS_H OL S**	87.7 ± 4.6	167 ± 15	166.5 ± 1.6	-32.1 ± 1.6	171.8 ±0.4	75.1 ± 5.2
**CS_H KL S**	90.9 ± 5.2	173 ± 7	168.4 ± 0.8	-32.1 ± 4.8	172.5 ±0.9	76.5 ± 3.7
**CS_L S**	89.2 ± 0.5	163 ± 1	160.2 ± 1,0	-36.0 ± 1.8	166.9 ± 2.2	71.8 ± 1.9
**CS_L OL S**	91.1 ± 3.3	145 ± 12	159.8 ± 0.3	-27.5 ± 0.3	164.8 ± 0.1	66.7 ± 1.2
**CS_L KL S**	92.3 ± 2.7	161 ± 5	162.3 ± 1.9	-30.8 ± 0*	169.0 ± 0.1	68.8 ± 0*

3.3. Isothermal plots of the real part of the conductivity (σ‘)

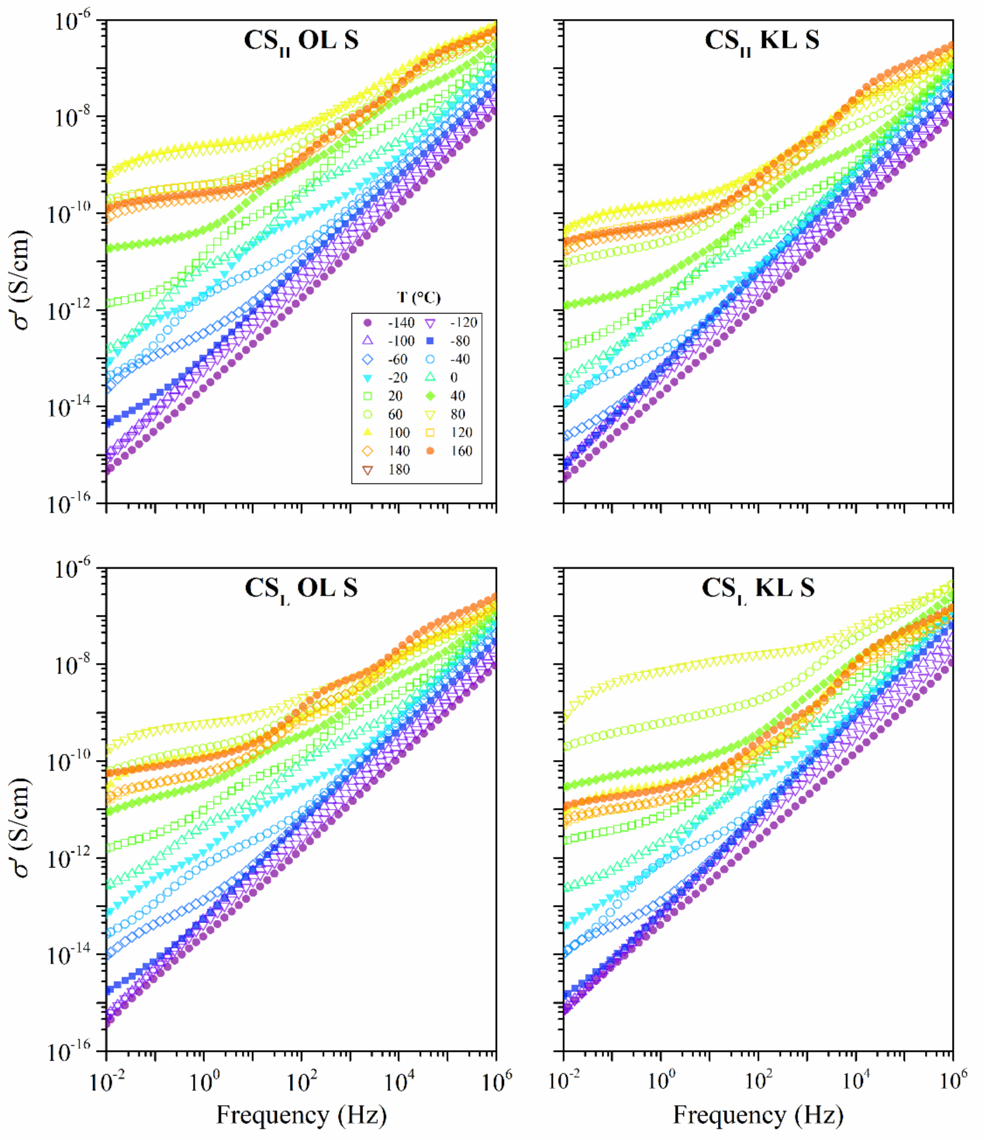

Figure S4: Real part of the conductivity (*σ'*) of the chitosan-lignin composites between 10^{-2} and 10^{5} Hz.

3.3. Bode Plots for proton conductivity determination

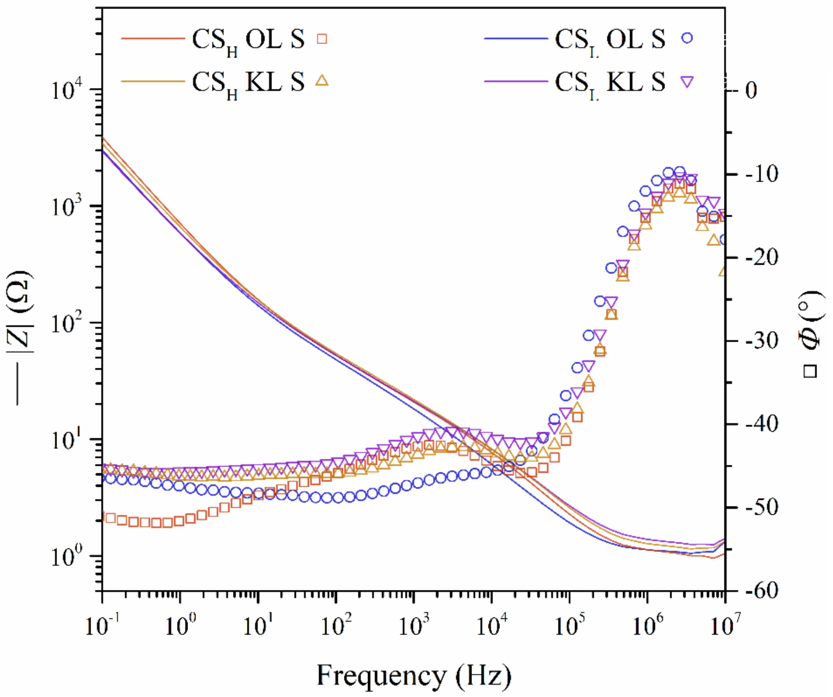

Figure S5: Bode plot of the chitosan-lignin composites at 60 °C including the modulus of impedance (|*Z*|) and phase angle (*Φ*)
